# Protective Role of Ginsenoside F1-Enriched Extract (SGB121) in Metabolic Dysfunction-Associated Fatty Liver Disease (MAFLD)

**DOI:** 10.3390/nu17233693

**Published:** 2025-11-25

**Authors:** Bo Yoon Chang, In Kim, Hyungmin Park, Sunchang Kim, Sung Yeon Kim

**Affiliations:** 1 Institute of Pharmaceutical Research and Development, College of Pharmacy, Wonkwang University, 460 Iksan-daero, Iksan-si 54538, Republic of Korea; 2 Cellonix Co. Ltd., 84, Gukgasikpum-ro, Wanggung-myeon, Iksan-si 54576, Republic of Korea; 3 Department of Biological Sciences, Korea Advanced Institute of Science and Technology, 291 Daehak-Ro, Yuseong-Gu, Daejeon 34141, Republic of Korea; 4 Intelligent Synthetic Biology Center, 291 Daehak-Ro, Yuseong-Gu, Daejeon 34141, Republic of Korea

**Keywords:** functional nutraceutical, ginsenoside F1-enriched extract (SGB121), metabolic dysfunction-associated fatty liver disease (MAFLD), antioxidant activity, insulin sensitization

## Abstract

**Introduction/Objectives**: Ginsenoside F1, a pharmacologically active saponin derived from Panax ginseng, exhibits diverse bioactivities, but its use is limited because it is difficult to purify and has high production costs. To overcome these challenges, a ginsenoside F1-enriched extract named SGB121 was developed. This study aimed to evaluate the therapeutic efficacy of SGB121 in a high-fat, high-carbohydrate (HFHC) diet-induced metabolic dysfunction-associated fatty liver disease (MAFLD) mouse model and to elucidate its mechanism of action using F1-based cellular assays. **Methods**: Male C57BL/6 mice (6 weeks old) were fed an HFHC diet to induce MAFLD and were treated with SGB121. Hepatic lipid accumulation, oxidative stress markers, and metabolic parameters were analyzed. In parallel, human hepatocellular carcinoma (HepG2) cells exposed to free fatty acids (FFAs) were used to assess oxidative stress and lipid accumulation. Mechanistic studies were conducted using purified F1 to examine adenosine monophosphate-activated protein kinase (AMPK) activation and related pathways. **Results**: SGB121 reduced hepatic lipid accumulation, malondialdehyde (MDA) levels, and fasting insulin while restoring glutathione (GSH) content and improving the homeostasis model assessment of insulin resistance (HOMA-IR) in MAFLD mice. In FFA-treated HepG2 cells, both SGB121 and F1 decreased reactive oxygen species (ROS), suppressed sterol regulatory element-binding protein 1 (SREBP1), enhanced peroxisome proliferator-activated receptor-α (PPARα) and β-oxidation, and restored insulin receptor substrate (IRS)/protein kinase B (Akt)/glucose transporter 2 (GLUT2) signaling. **Conclusions**: SGB121 ameliorates MAFLD and related metabolic dysfunction through antioxidant, lipid-regulating, and insulin-sensitizing actions, highlighting its potential as a safe multifunctional nutraceutical for MAFLD management.

## 1. Introduction

Metabolic dysfunction-associated fatty liver disease (MAFLD), recently redefined from the traditional term non-alcoholic fatty liver disease (NAFLD), represents a major metabolic liver disorder driven by Westernized dietary patterns such as an excessive intake of high-fat or high-carbohydrate foods [1,2,3]. This updated nomenclature emphasizes the disease’s close association with metabolic dysfunction rather than the mere exclusion of alcohol consumption [4]. MAFLD is characterized by hepatic lipid accumulation, insulin resistance, oxidative stress, and chronic inflammation, and its global prevalence continues to rise. The disease can progress from simple steatosis to metabolic dysfunction-associated steatohepatitis (MASH), fibrosis, and eventually hepatocellular carcinoma, yet effective pharmacological treatments remain limited [5]. This paradigm shift from NAFLD to MAFLD underscores the need for multi-targeted therapeutic strategies capable of modulating the complex metabolic, oxidative, and inflammatory networks underlying disease progression [6].

*Panax ginseng* has long been recognized for its diverse pharmacological actions, including anti-inflammatory, antioxidant, neuroprotective, and metabolic regulatory effects [7]. With the growing prevalence of metabolic disorders such as obesity, insulin resistance, and fatty liver disease worldwide, ginseng and its bioactive components have gained renewed attention as promising agents for metabolic health modulation. Recent advances in extraction and enzymatic conversion techniques have enabled the enrichment of specific ginsenosides, facilitating their development as standardized functional natural products with reproducible efficacy [8,9]. Among them, ginsenoside F1, a rare protopanaxatriol (PPT)-type minor ginsenoside [10], has emerged as a key compound of clinical interest, owing to its structural simplicity, superior bioavailability, and broad pharmacological activities [11].

Re and Rg1 are the primary precursors of F1, yet they exhibit minimal absorption in the upper gastrointestinal tract and require microbial β-glucosidase-mediated deglycosylation in the colon to generate F1, the bioactive form [11,12]. Because this microbial conversion depends heavily on individual gut microbiota composition, enzymatic capacity, and gastrointestinal pH, the amount of F1 produced by orally ingested precursors varies greatly among individuals. Such variability leads to inconsistent systemic exposure and makes it difficult to achieve predictable therapeutic effects [13]. Therefore, administering F1 directly, rather than relying on its microbial conversion from Re or Rg1, may provide a more reliable and consistent pharmacological outcome.

To overcome these limitations, a ginsenoside F1-enriched extract, SGB121, was developed by enzymatically converting Rg1 into F1 with β-glucosidase, followed by fractionation and purification to achieve F1 levels exceeding 5 mg/g [9]. This formulation enables a direct and consistent oral delivery of bioactive F1, independent of microbial metabolism, thereby improving pharmacokinetic uniformity and therapeutic reliability. Prior experimental and clinical have provided supporting evidence that F1 exhibits multiple beneficial effects, including anti-inflammatory [8], anti-atherosclerotic [14], neuroprotective [15], anticancer [16], and anti-aging [17] properties, through the modulation of AMP-activated protein kinase (AMPK), phosphatidylinositol 3-kinase/protein kinase B (PI3K/Akt), nuclear factor erythroid 2-related factor 2 (Nrf2), and Toll-like receptor 4 (TLR4) signaling pathways [18,19]. As demonstrated in our previous studies [17], the administration of SGB121 to mice fed a high-fat diet (HFD) significantly attenuated body weight gain and preserved hippocampal neuronal integrity without altering food intake, suggesting an intrinsic metabolic regulatory mechanism independent of caloric restriction. Given the rising global burden of metabolic dysfunction-associated fatty liver disease (MAFLD), which affects over one-quarter of the adult population and is tightly linked to obesity, insulin resistance, and cardiovascular morbidity, these findings suggest potential translational relevance for SGB121 as a preventive or adjunct therapeutic intervention.

Therefore, the present study aimed to elucidate the therapeutic potential and underlying mechanisms of SGB121 in the context of MAFLD. Specifically, it was designed with two major objectives: first, to evaluate the in vivo efficacy of SGB121 in a high-fat, high-carbohydrate (HFHC) diet-induced MAFLD mouse model by assessing hepatic steatosis, serum liver enzyme activity, oxidative stress, and histopathological alterations and, second, to investigate the cellular mechanisms of its principal bioactive component, ginsenoside F1, in free fatty acid (FFA)-challenged human hepatocellular carcinoma (HepG2) cells, with a particular focus on lipid metabolism, antioxidant defense, and insulin signaling pathways.

Together, these integrated in vivo and in vitro analyses provide a comprehensive understanding of the metabolic regulatory potential of SGB121 and support its development as a clinically relevant functional natural product for the prevention and management of MAFLD.

## 2. Materials and Methods

### 2.1. Chemicals and Reagents

Ginsenoside F1 (purity ≥ 98%, Cat. No. HY-N0598, CAS No. 53963-43-2) was purchased from MedChemExpress (MCE, Monmouth Junction, NJ, USA) and used for in vitro experiments. SGB121, a ginsenoside F1-enriched extract, was prepared from commercial ginseng extract (Daedong Korean Ginseng Co., Ltd., Geumsan, Buyeo, Republic of Korea) via the β-glucosidase (EC 3.2.1.21)-mediated enzymatic conversion of ginsenoside Rg1, followed by purification and lyophilization, as described in Section 2.2.

Oleic acid, palmitic acid, bovine serum albumin (BSA), paraformaldehyde, isopropanol, Oil Red O (ORO) dye, dimethyl sulfoxide (DMSO), and 3-(4,5-dimethylthiazol-2-yl)-2,5-diphenyltetrazolium bromide (MTT) were obtained from Sigma-Aldrich (St. Louis, MO, USA). The 2,2-diphenyl-1-picrylhydrazyl (DPPH) radical and methanol (high-performance liquid chromatography [HPLC] grade) were purchased from Wako Pure Chemical Industries, Ltd. (Osaka, Japan). A superoxide dismutase (SOD) activity assay kit was obtained from Dojindo Molecular Technologies (Kumamoto, Japan). Cell culture reagents, including Dulbecco’s Modified Eagle Medium (DMEM), fetal bovine serum (FBS), penicillin/streptomycin (Pen/Strep), and non-essential amino acids (NEAA), were purchased from Gibco (Thermo Fisher Scientific, Waltham, MA, USA). Primary antibodies against AMPK, phospho-AMPK, PPARα, SREBP1, ACC, FAS, CPT, IRS, Akt, GLUT2, and β-actin, as well as horseradish peroxidase (HRP)-conjugated secondary antibodies, were purchased from Cell Signaling Technology (Danvers, MA, USA) or Abcam (Cambridge, UK).

Unless otherwise stated, all other chemicals and reagents were of analytical grade, and deionized water was purified using a Milli-Q system (Millipore, Billerica, MA, USA).

### 2.2. Preparation of Ginsenoside SGB121

SGB121 was prepared from commercial ginseng extract (Daedong Korean Ginseng Co., Ltd., Republic of Korea) using an enzymatic conversion method previously described by Cui et al. [9]. The resulting extract was lyophilized and analyzed using HPLC, confirming the presence of ginsenoside F1 at approximately 0.5% (5 mg/g) of the total content (Table S1 and Figures S1 and S2).

### 2.3. Animal Tests

#### 2.3.1. Experimental Design for Animal Study

Five-week-old male C57BL/6J mice were purchased from Charles River Laboratories (Seoul, Republic of Korea) and housed under controlled environmental conditions (temperature 20 ± 2 °C and humidity 50 ± 10%) with a 12 h light/dark cycle. The mice were kept in polycarbonate cages (3–4 animals per cage) with free access to food and water (ad libitum). All animals underwent a one-week acclimation period prior to the start of the experiment.

Experimental group allocation was performed using a computer-based random number generator, ensuring randomization and minimizing group bias. To reduce potential confounding factors, cage positions were rotated weekly, and the order of treatment and measurement was kept consistent across all groups. The principal investigator and animal caretakers were aware of group assignments for dosing and animal welfare management; however, outcome evaluation and data analysis were conducted in a blinded manner to minimize bias. The mice were randomly assigned to the following five groups (*n* = 8 per group): (1) normal diet (NOR), (2) high-fat, high-carbohydrate diet control (CON), (3) HFHC + metformin 150 mg/kg (MET), (4) HFHC + SGB121 100 mg/kg, and (5) HFHC + SGB121 200 mg/kg.

The dosing levels of SGB121 were determined based on preliminary screening experiments demonstrating significant metabolic improvement at 200 mg/kg. The selected doses were designed not to exceed an equivalent intake of 1 g/kg when converted to a human equivalent dose, thereby ensuring safety and physiological relevance. A lower dose of 100 mg/kg was additionally included to assess dose–response relationships.

The normal diet consisted of the Teklad Global 18% Protein Rodent Diet (2018S; Envigo, Indianapolis, IN, USA), and the HFHC diet consisted of a DIO (diet-induced obesity) Purified Diet providing 60% of the total energy from fat (D12091402; Research Diets, New Brunswick, MO, USA) (Supplementary Materials Table S2). All animals were fed ad libitum for 12 weeks. SGB121 was administered orally (p.o.) five times per week throughout the experimental period, and metformin served as a positive control. At the end of the study, the mice were fasted for 12 h before sacrifice.

The experimental unit was defined as an individual animal. The sample size of eight mice per group was determined based on previous studies demonstrating statistically significant differences in similar diet-induced models while adhering to the ethical principle of minimizing animal use. No a priori power calculation was performed, as the parameters were already included in the approved Institutional Animal Care and Use Committee (IACUC) protocol.

Exclusion criteria were predefined as a body weight loss greater than 20% of the group mean according to the IACUC-approved protocol; however, no animals met these criteria during the experiment. All animals were monitored daily for health conditions and abnormal behaviors. Humane endpoints were established in accordance with the guidelines of the IACUC of Wonkwang University (approval number: WKU19-04).

All experimental procedures were conducted in compliance with the ARRIVE 2.0 guidelines and the institutional ethical standards of Wonkwang University to ensure reproducibility and animal welfare.

#### 2.3.2. Measurement of Body Weight and Composition

Body weights were measured weekly using a calibrated digital balance to monitor growth and health status throughout the study period. Body composition, including total fat mass and lean mass, was assessed using dual-energy X-ray absorptiometry (DEXA) with a full-body scanner (InAlyzer, Medikors, Seongnam, Republic of Korea). Prior to the scan, the animals were anesthetized with isoflurane (2–3% in oxygen, inhalation anesthetic) delivered via a nose cone to ensure immobilization and minimize stress. Each animal was placed in a standardized supine position (dorsal recumbency) on the scanner bed, with their limbs gently extended and aligned to maintain a consistent body posture. Scanning was performed in accordance with the manufacturer’s instructions, and data were analyzed using the accompanying InAlyzer software, version 24.1 (Medikors, Seongnam, Republic of Korea). The resulting parameters, including total body fat mass and lean mass, were recorded as both absolute values (g) and percentages of the total body weight.

#### 2.3.3. Measurement of Organ Weight

After completion of all dietary interventions and SGB121 administration, blood samples were collected from the abdominal vena cava under isoflurane anesthesia. The liver, perirenal fat, and epididymal fat tissues were then excised, and their wet weights were immediately measured (g). Each organ weight was normalized to the corresponding body weight of the individual animal to calculate the relative organ weight (%), which is expressed as the percentage of organ weight to body weight [20].

#### 2.3.4. Measurement of Hepatic Malondialdehyde (MDA) Levels

Liver tissue (approximately 0.1 g) was homogenized in 1 mL of ice-cold 1.15% potassium chloride (KCl) solution. The homogenate was centrifuged at 10,000 revolutions per min (RPM) for 10 min at 4 °C, and the supernatant was collected. To 200 µL of the supernatant, 200 µL of 20% trichloroacetic acid (TCA) and 200 µL of 0.67% thiobarbituric acid (TBA) in 0.1 N hydrochloric acid (HCl) were added. The mixture was vortexed, heated at 95 °C for 30 min in a water bath, and then rapidly cooled on ice. After cooling, the mixture was centrifuged at 3000 rpm for 10 min to remove precipitated proteins. The absorbance of the supernatant was measured at 532 nm using a spectrophotometer. Malondialdehyde (MDA) levels were calculated using a standard curve prepared with 1,1,3,3-tetramethoxypropane and are expressed as nmol MDA per gram of liver tissue, according to the thiobarbituric acid reactive substance (TBARS) method [21].

#### 2.3.5. Measurement of Hepatic Total Glutathione (GSH)

The reduced glutathione (GSH) concentrations were determined based on GSH oxidation with 5,5′-dithiobis-(2-nitrobenzoic acid) (DTNB), and the oxidized glutathione (GSSG) concentrations were determined enzymatically using glutathione reductase after inhibiting GSH oxidation with 2-vinylpyridine [22]. The reaction was monitored kinetically by measurinming the absorbance at 412 nm over a 1 min interval.

#### 2.3.6. Measurement of Hepatic and Serum Triglyceride and Cholesterol Levels

A total of 0.1 g of liver tissue was homogenized in 1 mL of ice-cold homogenization buffer consisting of 1.15% potassium chloride (KCl), 50 mM tris(hydroxymethyl)aminomethane (Tris), and 1 mM ethylenediaminetetraacetic acid (EDTA) (pH 7.4). The homogenate was centrifuged at 12,000 rpm for 10 min at 4 °C, and the supernatant was collected for analysis. Blood samples were collected from the abdominal vena cava and centrifuged at 3000 rpm for 20 min, and the resulting serum was separated for use. The hepatic and serum levels of triglycerides (TGs) and total cholesterol (TC) were quantified using enzymatic colorimetric assay kits (Biovision, Milpitas, CA, USA), following the manufacturer’s instructions. Absorbance was measured at 570 nm using a microplate reader. Lipid concentrations were calculated from standard curves and are expressed as mg/g tissue for liver samples and mg/dL for serum samples [23,24].

#### 2.3.7. Measurement of Serum AST and ALT Activities

Serum from the collected blood samples was used for the determination of aspartate amino transferase (AST) and alanine aminotransferase (ALT) levels according to the method described by Reitman and Frankel [25]. The substrates in the reaction were α-ketoglutaric acid and L-aspartate for aspartate aminotransferase (AST), and α-ketoglutaric acid and L-alanine for alanine aminotransferase (ALT). The mixture was then added with 2,4-dinitrophenylhydrazine (DNPH) and incubated at room temperature. Sodium hydroxide (NaOH) was subsequently added, and the color intensity was measured at 540 nm using a spectrophotometer.

#### 2.3.8. Measurement of Fasting Blood Glucose, Insulin, and HOMA-IR

At the end of the experimental period, the animals were fasted for 12 h prior to sacrifice. Blood samples were collected via the abdominal vena cava under anesthesia. Fasting blood glucose levels were measured in whole blood using a glucometer (OneTouch Ultra, Inverness Medical Ltd., Waltham, MA, USA). Serum insulin concentrations were determined using a commercially available enzyme-linked immunosorbent assay (ELISA) kit (Invitrogen, Thermo Fisher Scientific, Waltham, MA, USA), according to the manufacturer’s instructions. The homeostasis model assessment of insulin resistance (HOMA-IR) was calculated using the following formula [26]:

HOMA-IR = (fasting glucose (mg/dL) × fasting insulin (μIU/mL))/405

These parameters were used to evaluate glucose homeostasis and insulin sensitivity in each group.

#### 2.3.9. Measurement of Hepatic Lipid Accumulation Using Histological Analysis

The excised liver tissues were immediately dehydrated using the sucrose infiltration method and subsequently embedded for cryosectioning. Frozen liver sections with a thickness of 5 μm were prepared and subjected to Oil Red O (ORO) staining to evaluate hepatic lipid accumulation, followed by hematoxylin counterstaining for nuclear visualization [27]. The stained sections were observed using an optical microscope (Leica DM2000, Leica Microsystems, Wetzlar, Germany) at 200× magnification, and three representative fields were captured per sample. A quantitative analysis of hepatic lipid accumulation was performed using ImageJ software, version 1.54p (National Institutes of Health [NIH], Bethesda, MD, USA) by applying color thresholding to isolate the red-stained (Oil Red O-positive) regions. The ORO-positive area was calculated as a percentage of the total tissue area.

### 2.4. Measurement of DPPH and Superoxide Radical Scavenging Activities of F1 and SGB121 Under Cell-Free Conditions

The DPPH and superoxide radical scavenging activities of SGB121 or F1 were evaluated under cell-free conditions using a DPPH assay (0.1 mM in methanol, mixed with the sample in a 1:1 ratio and measured at 520 nm after 30 min in the dark) [28] and a commercially available SOD assay kit (Dojindo Molecular Technologies, Kumamoto, Japan) [29], respectively; vitamin C (50 µM) and Trolox (200 µg/mL) were used as positive controls for the DPPH and SOD assays, respectively.

### 2.5. Cell Line

HepG2 cells were obtained from the American Type Culture Collection (ATCC, Manassas, VA, USA) and cultured in DMEM (Gibco, Thermo Fisher Scientific, Waltham, MA, USA) supplemented with 10% FBS (Gibco, Thermo Fisher Scientific, Waltham, MA, USA) and 1% Pen/Strep (Gibco, Thermo Fisher Scientific, Waltham, MA, USA) at 37 °C in a humidified atmosphere containing 5% CO_2_. The medium was replaced every 2–3 days, and the cells were subcultured at approximately 80–90% confluence using 0.05% trypsin-EDTA.

### 2.6. Measurement of Cell Viability Following F1 and SGB121 Treatment in HepG2 Cells Using the MTT Assay

Cell viability was assessed using the MTT assay [30]. Briefly, human hepatocellular carcinoma (HepG2) cells were seeded in 96-well plates at a density of 1 × 10^4^ cells/well and allowed to attach for 18 h. The cells were then treated with various F1 or SGB121 concentrations for 24 h. After treatment, 100 µL of MTT solution (1 mg/mL in phosphate-buffered saline [PBS]) was added to each well and incubated for 2 h at 37 °C. The resulting formazan crystals were dissolved in 100 µL of DMSO, and absorbance was measured at 570 nm using a microplate reader. Cell viability is expressed as a percentage relative to that of the untreated control group. Based on the results of the MTT assay, subsequent experiments were conducted using F1 or SGB121 concentrations that did not induce significant cytotoxicity.

### 2.7. Assessment of FFA-Induced Oxidative Stress and Lipid Accumulation

HepG2 cells were treated with a mixture of FFAs composed of oleic acid and palmitic acid at a 2:1 molar ratio, either in the presence or absence of F1 or SGB121. The total FFA concentration was adjusted to 1.0 mM (0.67 mM oleic acid + 0.33 mM palmitic acid), a dose commonly used to induce hepatic steatosis and oxidative stress in vitro. The FFA mixture was prepared by conjugating fatty acids with fatty acid-free bovine serum albumin (BSA; Sigma-Aldrich, St. Louis, MO, USA) in serum-free DMEM and applied to the cells for 24 h [31]. Following treatment, intracellular reactive oxygen species (ROS) levels and the intracellular lipid content were measured using DCFH-DA staining and Oil Red O staining, respectively, as described below.

#### 2.7.1. Measurement of Intracellular ROS

HepG2 cells with FFA-induced oxidative stress were assessed for intracellular reactive oxygen species (ROS) levels using the oxidation-sensitive fluorescent probe 2′,7′-dichlorodihydrofluorescein diacetate (DCFH-DA) [32]. After treatment, the cells were incubated with 10 µM DCFH-DA at 37 °C for 30 min in the dark. The fluorescence intensity was measured using a microplate reader (BioTek, Winooski, VT, USA) at an excitation wavelength of 490 nm and an emission wavelength of 525 nm. All measurements were conducted in black, flat-bottom 96-well plates to minimize background fluorescence and improve signal accuracy. Metformin was used as a positive control to confirm the assay’s sensitivity in detecting reductions in ROS levels.

#### 2.7.2. Quantification of Lipid Accumulation

HepG2 cells with FFA-induced lipid accumulation were treated with or without F1 or SGB121 for 24 h. After incubation, the cells were washed twice with phosphate-buffered saline (PBS) to remove residual FFAs and fixed with 4% paraformaldehyde for 1 h at room temperature. The fixed cells were washed three times with PBS and stained with freshly prepared Oil Red O solution for 20 min to visualize intracellular lipid droplets. Unbound dye was removed by rinsing with distilled water, and the retained dye was subsequently eluted using 100% isopropanol. The absorbance of the extracted dye was measured at 510 nm using a microplate reader [33]. Lipid accumulation was quantified by the absorbance value, which is expressed as a percentage relative to that of the untreated control group.

#### 2.7.3. Measurement of Gene Expression Levels in HepG2 Cells Using Quantitative Real-Time PCR

Total RNA was extracted from HepG2 cells using a commercially available RNA extraction kit, according to the manufacturer’s instructions. For cell-based experiments, RNA was isolated from at least three independently cultured plates (biological replicates), and each experiment was repeated to ensure reproducibility. In the animal studies, RNA was extracted individually from the liver tissue of all animals in each group, and data were analyzed collectively [34]. Quantitative real-time PCR was performed using a TaqMan RNA-to-CT 1-Step Kit (Applied Biosystems, Foster City, CA, USA) in a 20 µL reaction volume containing template RNA, gene-specific probes, reverse transcriptase, and DNA polymerase. Amplification and detection were carried out using the StepOne Real-Time PCR System (Applied Biosystems), and relative gene expression was calculated using the ^2^ΔΔCT method. The following pre-designed assays specific for human genes were used: sterol regulatory element-binding protein-1c (SREBP-1c; Hs01088691_m1), fatty acid synthase (FAS; Hs01005622_m1), acetyl-CoA carboxylase (ACC; Hs01046047_m1), peroxisome proliferator-activated receptor alpha (PPARα; Hs00947536_m1), heme oxygenase-1 (HO-1; Hs01110250_m1), nuclear factor erythroid 2-related factor 2 (NRF2; Hs00975961_g1), glutamate–cysteine ligase catalytic subunit (GCLC; Hs00155249_m1), glucose transporter 2 (GLUT2; Hs01096905_m1), and glyceraldehyde-3-phosphate dehydrogenase (GAPDH; Hs02786624_g1). Relative mRNA expression levels were calculated using the ^2^ΔΔCt method, and the results are expressed as fold changes relative to those of the normal control group.

#### 2.7.4. Measurement of Target Protein Expression in HepG2 Cells

HepG2 cells were lysed in radioimmunoprecipitation assay (RIPA) buffer supplemented with a protease and phosphatase inhibitor cocktail. Prior to lysis, both cell pellets and homogenized liver tissues were disrupted using a probe-type ultrasonic homogenizer to ensure efficient protein extraction. The lysates were incubated on ice for 30 min and then centrifuged at 13,000 rpm for 25 min at 4 °C. Nuclear and cytoplasmic proteins were separated using a commercial fractionation kit (NE-PER™, Thermo Fisher) following the manufacturer’s protocol [35]. Briefly, the samples were treated with cytoplasmic extraction reagents and centrifuged to collect the cytoplasmic fraction, and the remaining pellet was extracted with nuclear extraction reagent to obtain the nuclear fraction. The resulting supernatants were collected, and protein concentrations were quantified. Equal amounts of protein (20 µg) were separated by SDS-polyacrylamide gel electrophoresis and transferred to PVDF membranes. Membranes were blocked in 5% skim milk or BSA for 1 h at room temperature, followed by overnight incubation at 4 °C with primary antibodies specific to the target proteins. The following primary antibodies from Santa Cruz Biotechnology (Santa Cruz, CA, USA) were used: AKT (B-1) (sc-5298, mouse monoclonal), AMPKα1/2 (D-6) (sc-74461, mouse monoclonal), CPT1 (E-7) (sc-577360, mouse monoclonal), FAS (G-9) (sc-74540, mouse monoclonal), GLUT2 (C-10) (sc-518022, mouse monoclonal), HO-1 (A-3) (sc-136960, mouse monoclonal), IRS1 (sc-559, rabbit polyclonal), NQO1 (A-5) (sc-376023, mouse monoclonal), Nrf2 (A-10) (sc-365949, mouse monoclonal), p-ACCα (F-2) (sc-271965, mouse monoclonal), p-AKT (Ser473) (sc-514032, mouse monoclonal), p-AMPKα (Thr172) (sc-33524, rabbit polyclonal), p-IRS1 (Ser307) (sc-33929, rabbit polyclonal), PPARα (H-2) (sc-398394, mouse monoclonal), and SREBP-1 (A-4) (sc-365513, mouse monoclonal). Secondary antibodies and detection were performed in accordance with the manufacturer’s recommendations. Protein bands were detected using an enhanced chemiluminescence (ECL) detection kit and visualized using the ChemiDoc Go Imaging System (Bio-Rad Laboratories, Hercules, CA, USA), which is a compact, high-sensitivity benchtop imager equipped with advanced CMOS digital imaging technology. β-actin was used as a loading control. Band intensities were quantified using ImageJ software (NIH, Bethesda, MD, USA).

### 2.8. Statistical Analysis

Statistical calculations were performed using GraphPad Prism software, version 10.0 (GraphPad Software Inc., San Diego, CA, USA). A one-way ANOVA, followed by Tukey’s multiple comparison test, was used to determine statistical significance among groups. The results are expressed as the mean ± SE, and differences were considered statistically significant at *p* < 0.05. Different letters indicate statistically significant differences between groups.

## 3. Results

### 3.1. Animal Experiment for SGB121

#### 3.1.1. Effects of SGB121 on Body Weight of HFHC Diet-Induced MAFLD Mice

**Figure 1:**
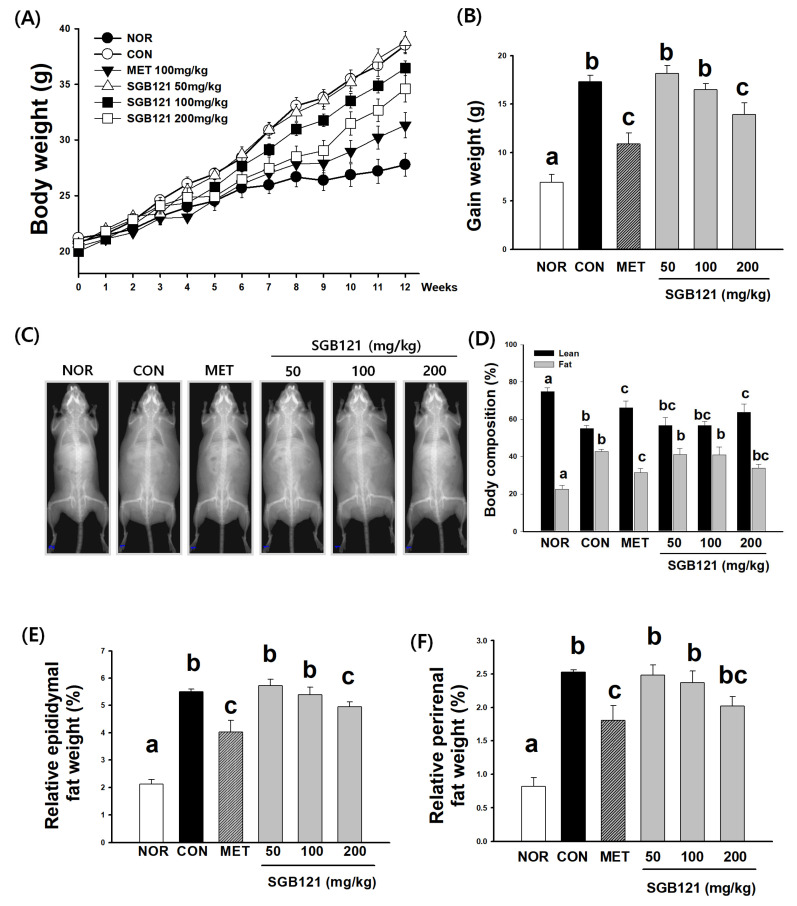
Animal experiment examining the effects of SGB121 on pathophysiological parameters in high-fat, high-carbohydrate (HFHC) diet-induced metabolic-associated fatty liver disease (MAFLD) mice. C57BL/6 mice were fed an HFHC diet for 12 weeks and orally administered SGB121 (50, 100, or 200 mg/kg) once daily during the feeding period. Metformin (MET, 150 mg/kg) was used as a positive control. Normal (NOR) mice received a standard chow diet, and HFHC (CON) mice were administered vehicle only. (**A**) Body weight changes over 12 weeks, (**B**) total weight gain, (**C**) representative dual-energy X-ray absorptiometry (DXA) images, (**D**) body composition analysis, (**E**) relative epididymal fat weight, and (**F**) relative perirenal fat weight (sum of perirenal, mesenteric, and retroperitoneal fat depots). Data are expressed as the mean ± SE (*n* = 8). Statistical significance was determined using a one-way ANOVA, followed by Tukey’s multiple comparison test. Different superscript letters indicate statistically significant differences among groups (*p* < 0.05).

At the beginning of the experiment, the average body weight of the mice was 20.9 ± 0.4 g. After 12 weeks, the average body weight in the NOR group reached 27.8 ± 1.0 g, whereas that in the CON group markedly increased to 36.7 ± 1.1 g, confirming the diet’s obesity-inducing effect. In contrast, the administration of SGB121 effectively attenuated body weight gain compared with CON. Treatment with 50 mg/kg (38.8 ± 0.9 g) and 100 mg/kg SGB121 (36.9 ± 1.3 g) did not have significant effects on body weight compared with CON, whereas 200 mg/kg SGB121 markedly decreased body weight to 35.4 ± 1.3 g (3.7% reduction), showing a statistically significant difference relative to CON (Figure 1A,B).

#### 3.1.2. Reduction in Adiposity and Preservation of Lean Mass by SGB121

DEXA and gross imaging clearly demonstrated excessive fat accumulation in the CON group compared with the NOR group (Figure 1C). In contrast, SGB121 treatment reduced adiposity in a dose-dependent manner, with the most pronounced reductions observed at 100 and 200 mg/kg. A body composition analysis revealed a similar pattern, showing that the fat mass percentage was markedly elevated in the CON group (approximately 45% of total body weight) relative to the NOR group (approximately 25%) (Figure 1D). However, the administration of SGB121 at 100 and 200 mg/kg significantly decreased the fat mass ratio to 33% and 30%, respectively.

Lean mass also exhibited notable differences across groups. The CON group showed a significant reduction in lean mass compared with the NOR group, reflecting the characteristic metabolic lean loss induced by the HFHC diet. Although MET partially improved lean mass relative to CON, it did not fully recover it to NOR levels. Interestingly, lean mass in the groups receiving SGB121 treatment, particularly at 200 mg/kg, was significantly higher than in the CON group, forming distinct statistical groups compared with both the NOR and MET groups. These findings suggest that higher doses of SGB121 not only reduce fat accumulation but also contribute to the preservation or partial recovery of lean tissue.

As shown in Figure 1E, the relative epididymal fat weight was 2.1 ± 0.2% in NOR and increased to 5.1 ± 0.2% in CON. Compared with CON, SGB121 administration decreased the relative epididymal fat weight by 11.7% (50 mg/kg), 13.7% (100 mg/kg), and 13.2% (200 mg/kg).

MET reduced epididymal fat to 4.0 ± 0.4%. As shown in Figure 1F, the relative perirenal fat weight was 0.8 ± 0.1% in NOR and increased to 2.2 ± 0.1% in CON. SGB121 treatment reduced perirenal fat by 0.7% (50 mg/kg), 7.6% (100 mg/kg), and 12.7% (200 mg/kg) when compared with CON. MET lowered fat to 1.8 ± 0.2%.

These results (Figure 1E,F) indicate that SGB121 supplementation attenuated both perirenal and epididymal fat accumulation in CON, with the most pronounced effect observed at 200 mg/kg.

#### 3.1.3. Hepatoprotective Effects of SGB121 via Reduction of Lipid Accumulation and Oxidative Stress

**Figure 2:**
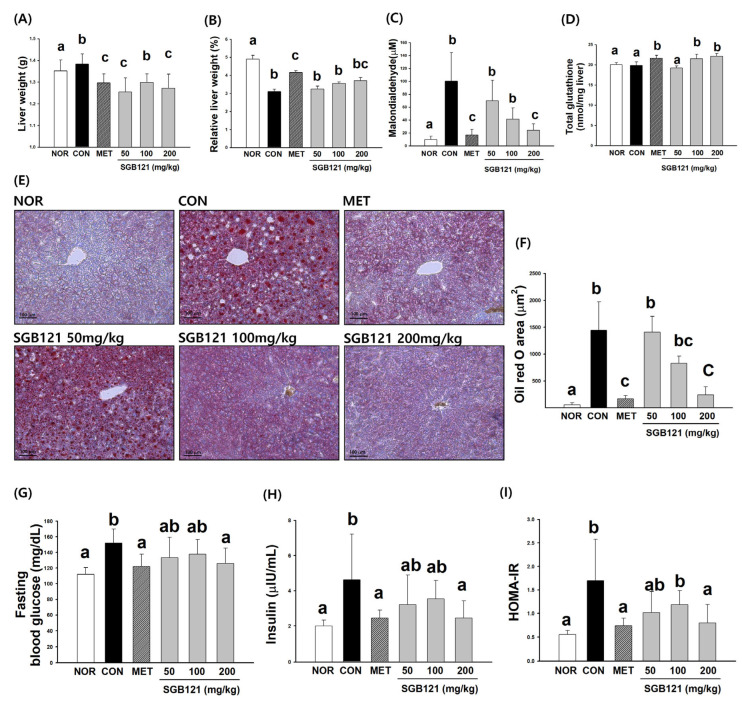
Effects of SGB121 on hepatic histology, oxidative stress, and metabolic parameters in high-fat, high-carbohydrate (HFHC) diet-induced MAFLD mice. C57BL/6 mice were fed an HFHC diet for 12 weeks and orally administered SGB121 (50, 100, or 200 mg/kg) once daily during the feeding period. Normal (NOR) mice were fed a standard chow diet, while HFHC (CON) mice received the HFHC diet with vehicle only. Metformin (MET, 150 mg/kg) was used as a positive control. (**A**) Absolute liver weight, (**B**) relative liver weight, (**C**) hepatic malondialdehyde (MDA) levels, and (**D**) total glutathione (GSH) content were measured to evaluate hepatic oxidative stress. (**E**) Representative liver sections stained with Oil Red O and hematoxylin (scale bar = 100 μm). Lipid droplets stained in red indicate hepatic fat accumulation. (**F**) Quantification of Oil Red O-positive areas analyzed using ImageJ software. (**G**) Fasting blood glucose levels, (**H**) serum insulin levels, and (**I**) homeostatic model assessment of insulin resistance (HOMA-IR) values. Data are expressed as the mean ± SE (*n* = 8). Statistical significance was determined using a one-way ANOVA, followed by Tukey’s multiple comparison test. Different superscript letters indicate statistically significant differences among groups (*p* < 0.05).

As shown in Figure 2A, the absolute liver weight in the CON group significantly increased compared with that in the NOR group, whereas the administration of 200 mg/kg SGB121 resulted in a significant reduction relative to CON. Consistently, the relative liver weight was 4.9 ± 0.2% in the NOR group and markedly decreased to 3.1 ± 0.1% in the CON group. SGB121 treatment increased the relative liver weight by 4.4%, 14.6%, and 19.6% at doses of 50, 100, and 200 mg/kg, respectively, demonstrating a dose-dependent trend toward recovery. MET also restored the relative liver weight by 33.7% compared with CON (Figure 2B). A histological examination (Figure 2E) revealed extensive lipid droplet accumulation in CON, while metformin and SGB121 supplementation improved hepatic morphology. The improvement was most evident in the 200 mg/kg SGB121 group.

As shown in Figure 2C, hepatic MDA levels were 9.8 ± 5.1 μM in the NOR group and markedly elevated to 100.2 ± 44.3 μM in the CON group. SGB121 administration decreased MDA by 30.3%, 58.7%, and 75.8% at 50, 100, and 200 mg/kg, respectively, indicating a dose-dependent and significant reduction, and MET reduced MDA by 83.2% compared with CON. As shown in Figure 2D, hepatic GSH levels were 20.1 ± 0.5 nmol/mg in the NOR group and slightly decreased to 19.8 ± 0.9 nmol/mg in the CON group. SGB121 treatment increased GSH levels by 8.4% and 11.5% at 100 and 200 mg/kg, respectively, showing a trend toward dose-dependent improvement, and metformin elevated GSH by 9.0% compared with CON.

These findings indicate that SGB121 supplementation significantly attenuated hepatic lipid peroxidation and improved antioxidant capacity, accompanied by a partial recovery of liver weight and morphology in the HFHC diet-induced MAFLD mice.

#### 3.1.4. Improvement in Glucose Homeostasis and Insulin Sensitivity by SGB121

As shown in Figure 2G, fasting blood glucose was 112.0 ± 8.8 mg/dL in the NOR group and increased to 151.9 ± 18.1 mg/dL in the CON group (1.36-fold increase vs. NOR). MET treatment reduced blood glucose by 19.6% compared with CON, while SGB121 decreased blood glucose by 12.2%, 9.8%, and 17.2% at 50, 100, and 200 mg/kg, respectively.

Serum insulin was 2.0 ± 0.3 µU/mL in the NOR group and increased to 4.6 ± 2.6 µU/mL in the CON group (2.3-fold increase vs. NOR). MET reduced insulin levels by 46.9% compared with CON, while SGB121 decreased insulin levels by 30.6% at 50 mg/kg, showed a slight reduction of 23.5% at 100 mg/kg, and achieved a marked reduction of 46.9% at 200 mg/kg (Figure 2H).

HOMA-IR was 0.5 ± 0.1 in the NOR group and increased to 1.7 ± 0.9 in the CON group (3.07-fold increase vs. NOR). MET treatment reduced HOMA-IR by 56.5% compared with CON, while SGB121 decreased HOMA-IR by 40.1%, 30.2%, and 53.0% at 50, 100, and 200 mg/kg, respectively (Figure 2I).

These results demonstrate that SGB121 supplementation significantly reduced fasting blood glucose, insulin, and HOMA-IR in the HFHC diet-induced MAFLD mice, with the strongest effect observed at 200 mg/kg, comparable to MET.

#### 3.1.5. Improvement in Lipid Metabolism and Liver Enzyme Levels by SGB121

As presented in Table 1, the TG levels were 6.7 ± 0.6 mg/g liver in NOR and markedly increased to 62.8 ± 7.6 mg/g liver in CON. SGB121 supplementation reduced hepatic TG levels by 22.9%, 20.6%, and 55.5% at 50, 100, and 200 mg/kg, respectively, exhibiting a dose-dependent decline, while MET decreased TG levels by 41.7% compared with CON. The serum TG levels were 46.4 ± 4.9 mg/dL in NOR and increased to 101.5 ± 2.9 mg/dL in CON. Treatment with SGB121 lowered serum TG levels by 16.2%, 24.9%, and 24.5% at 50, 100, and 200 mg/kg, respectively, whereas MET reduced TG levels by 49.1% compared with CON. The TC was 3.0 ± 0.2 mg/g liver in NOR and elevated to 8.2 ± 1.1 mg/g liver in CON. SGB121 administration decreased the hepatic TC by 13.3%, 23.9%, and 54.2% at 50, 100, and 200 mg/kg, respectively, showing a dose-related improvement, and MET also reduced the TC by 37.0% compared with CON. The serum TC was 152.8 ± 5.7 mg/dL in NOR and rose to 242.3 ± 13.5 mg/dL in CON. SGB121 decreased the serum TC by 6.0%, 1.8%, and 15.8% at 50, 100, and 200 mg/kg, respectively, while MET lowered the TC by 11.0% compared with CON. ALT activity was 32.3 ± 4.5 U/mL in NOR and significantly increased to 61.0 ± 7.7 U/mL in CON. SGB121 reduced ALT activity by 7.7%, 14.0%, and 12.1% at 50, 100, and 200 mg/kg, respectively, whereas MET decreased ALT activity by 32.4% compared with CON. AST activity was 61.6 ± 6.7 U/mL in NOR and elevated to 91.3 ± 8.0 U/mL in CON. SGB121 supplementation decreased AST activity by 24.3%, 9.6%, and 22.0% at 50, 100, and 200 mg/kg, respectively, and MET reduced AST activity by 26.3% compared with CON.

### 3.2. SGB121 Enhances Antioxidant Capacity and Attenuates Lipid Accumulation in HepG2 Cells

**Figure 3:**
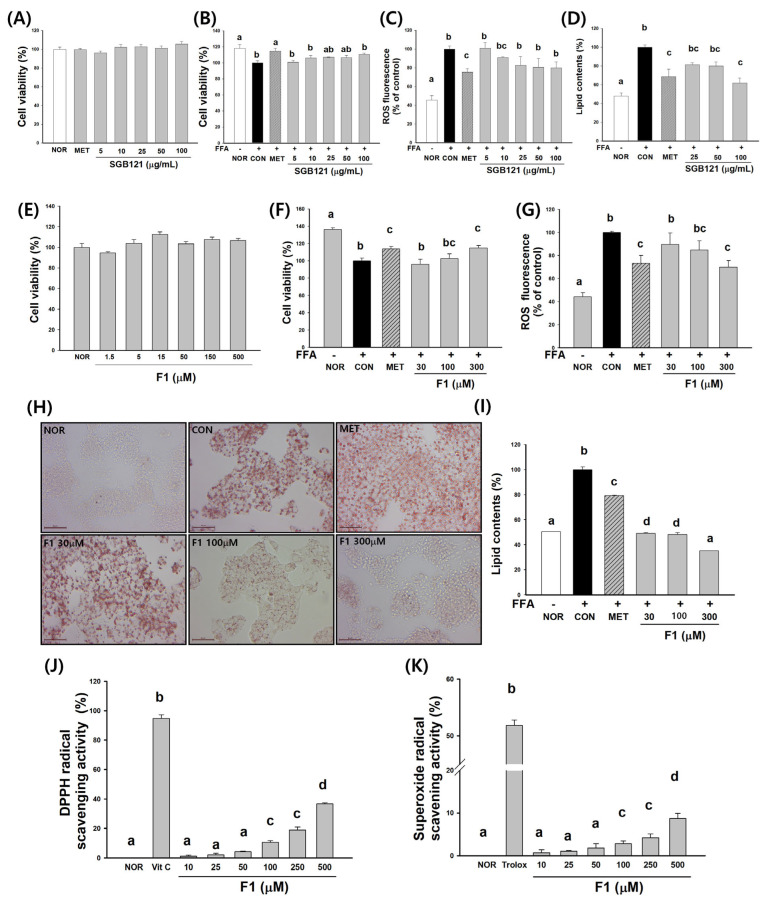
Protective effects of SGB121 and ginsenoside F1 against oxidative stress and lipid acumulation in HepG2 cells. HepG2 cells were treated with a mixture of free fatty acids (FFAs; oleic acid and palmitic acid, 2:1 ratio) to induce lipotoxicity. Normal (NOR) cells were cultured under basal conditions without FFA treatment, while FFA-treated control (CON) cells were exposed to FFAs without any extract or compound. Metformin (MET, 1 mM) was used as a positive control. (**A**) Cell viability of HepG2 cells treated with various SGB121 concentrations (5–200 µg/mL) under normal conditions. (**B**) Effects of SGB121 (5–100 µg/mL) on FFA-induced cytotoxicity. (**C**) Inhibition of FFA-induced intracellular reactive oxygen species (ROS) generation by SGB121 (5–100 µg/mL). (**D**) Suppression of FFA-induced lipid accumulation by SGB121 (25–100 µg/mL). (**E**) Cell viability of HepG2 cells treated with ginsenoside F1 (1.5–500 µM) under normal conditions. (**F**) Protective effect of F1 (30–300 µM) on FFA-induced cytotoxicity. (**G**) Inhibition of FFA-induced intracellular ROS levels by F1. (**H**) Oil Red O staining showing lipid accumulation in HepG2 cells; F1 treatment reduced lipid accumulation in a dose-dependent manner (scale bar = 100 µm). (**I**) Quantitative analysis of lipid content based on Oil Red O staining. (**J**) 2,2-Diphenyl-1-picrylhydrazyl (DPPH) radical scavenging activity of F1 (10–500 µM), with vitamin C (vit C) used as a positive control. (**K**) Superoxide radical scavenging activity of F1 (10–500 µM), with trolox used as a positive control. Data are presented as the mean ± SE (*n* = 3). Statistical significance was determined using a one-way ANOVA, followed by Tukey’s multiple comparison test. Different superscript letters indicate statistically significant differences among groups (*p* < 0.05).

To determine the appropriate concentration range of SGB121 for in vitro experiments, cell viability was assessed across a range of 5 to 100 µg/mL. At the highest concentration tested (200 µg/mL), cell viability was 81.1%, indicating a slight reduction but not a level indicative of significant cytotoxicity. Therefore, SGB121 was considered to have an acceptable safety margin, and subsequent experiments were conducted using concentrations up to 100 µg/mL (Figure 3A). Oxidative stress was induced by treatment with FFAs, resulting in a marked decrease in cell viability compared to NOR. However, pretreatment with SGB121 at a concentration of 25 µg/mL or higher significantly improved cell viability relative to the FFA-treated (CON) group, demonstrating a protective effect (Figure 3B).

Intracellular ROS levels, which were substantially elevated in CON, were significantly reduced following SGB121 treatment. A notable reduction in ROS was observed in cells treated with 50 and 100 µg/mL of SGB121 compared to CON, indicating effective attenuation of oxidative stress (Figure 3C).

In the lipid accumulation model, FFA-treated HepG2 cells showed substantial intracellular lipid build-up compared to the normal group. Treatment with SGB121 at concentrations of 25, 50, and 100 µg/mL resulted in a significant reduction in lipid accumulation relative to the FFA-only control group, with the greatest reduction observed at 100 µg/mL. These results suggest that SGB121 effectively inhibits hepatic lipid accumulation and exerts protective effects against steatosis (Figure 3D).

### 3.3. Ginsenoside F1 Enhances Antioxidant Capacity and Attenuates Lipid Accumulation in HepG2 Cells

To investigate the antioxidant and hepatoprotective effects of ginsenoside F1, a series of in vitro experiments were performed using HepG2 cells (Figure 3E–K).

#### 3.3.1. Cytotoxicity and Cell Viability

F1 treatment did not induce cytotoxicity at concentrations up to 500 µM, with cell viability consistently maintained above 90%, indicating favorable biocompatibility. Based on these findings, 300 µM was selected as the maximum concentration for subsequent in vitro experiments to balance efficacy and safety (Figure 3E).

#### 3.3.2. Protection Against Oxidative Stress

Exposure to free fatty acids (FFAs) significantly reduced cell viability compared to the normal group. Pretreatment with F1 at 150 and 300 µM markedly restored cell viability relative to the FFA-treated control group. Consistently, intracellular ROS levels quantified using DCFH-DA fluorescence were significantly elevated following FFA exposure but were significantly attenuated in a dose-dependent manner by F1 treatment, with the greatest reduction observed at 300 µM (Figure 3F,G).

#### 3.3.3. Inhibition of Lipid Accumulation

FFA-induced steatosis resulted in marked intracellular lipid accumulation in HepG2 cells, as visualized by Oil Red O staining under light microscopy (Figure 3H,I). Cells treated with FFAs exhibited numerous dense, red-stained lipid droplets within the cytoplasm, indicating the successful induction of steatosis. In contrast, cells treated with F1 at 100 and 300 µM showed a visible reduction in both the number and intensity of lipid droplets compared to the FFA-treated control group. These morphological findings were consistent with the quantitative data obtained via the elution of the retained dye, which confirmed a significant dose-dependent decrease in the lipid content in the F1-treated groups. These results suggest that F1 effectively mitigates lipid accumulation and may exert protective effects against hepatocellular steatosis.

#### 3.3.4. Radical Scavenging Activity

F1 demonstrated dose-dependent free radical scavenging activity under cell-free conditions (Figure 3J,K). In the DPPH assay, F1 significantly increased the radical scavenging capacity beginning at 50 µM, with the maximal effect observed at 500 µM, comparable to that of vitamin C. Similarly, in the superoxide radical scavenging assay, F1 exhibited significant activity at concentrations ≥100 µM, with the strongest effect at 500 µM, although it remained lower than that of the positive control, Trolox.

### 3.4. F1 Activates AMPK and Modulates Lipid Metabolism-Related Proteins

#### 3.4.1. Regulation of Lipid Metabolism by F1 Through AMPK Activation

**Figure 4:**
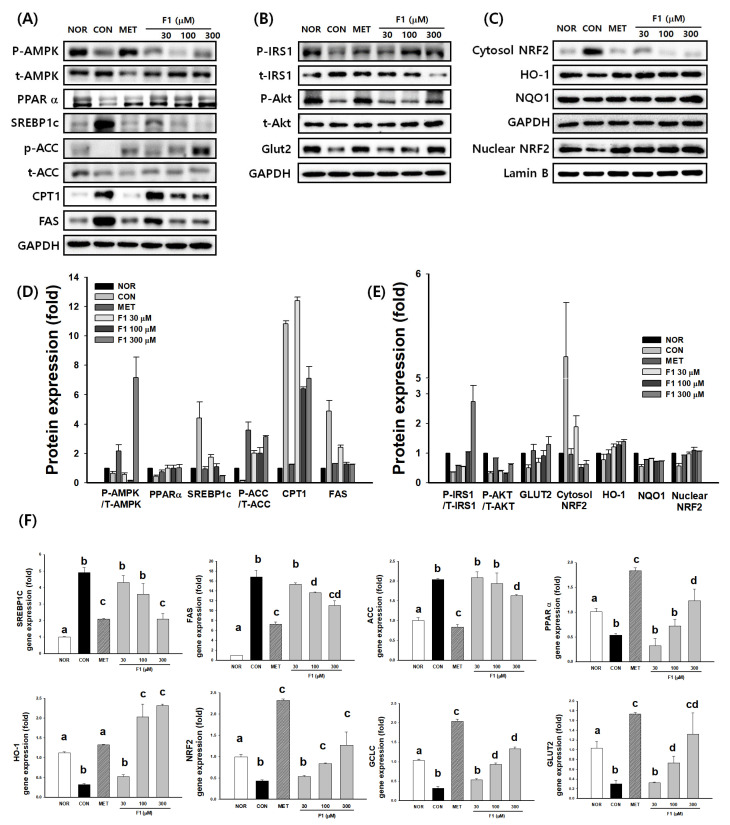
Effects of F1 on AMPK activation, insulin signaling, NRF2-mediated antioxidant responses, and gene expression related to lipid metabolism. HepG2 cells were treated with a mixture of free fatty acids (FFAs; oleic acid and palmitic acid, 2:1 ratio) to induce lipotoxicity. Normal control (NOR) cells were cultured under basal conditions without FFA treatment, while FFA-treated control (CON) cells were exposed to FFA without any extract or compound. Cells were treated with ginsenoside F1 (30, 100, or 300 μM) or metformin (MET, 1 mM) for 24 h. (**A**) Western blot analysis of proteins involved in AMPK signaling (p-AMPK, t-AMPK, PPARα, SREBP1c, p-ACC, t-ACC, CPT1, and FAS). (**B**) Insulin signaling (p-IRS1, t-IRS1, p-Akt, t-Akt, and GLUT2). (**C**) NRF2 signaling (cytosolic NRF2, nuclear NRF2, HO-1, and NQO1). GAPDH and Lamin B served as loading controls for cytosolic and nuclear proteins, respectively. (**D**,**E**) Bar graphs represent densitometric quantification of protein expression normalized to the respective total protein or loading control. (**F**) mRNA expression levels of SREBP1c, FAS, ACC, PPARα, HO-1, NRF2, GCLC, and GLUT2 determined by quantitative PCR (qPCR) after the indicated treatments. Data are presented as fold changes relative to the CON group (mean ± SE, *n* = 3). Statistical significance was determined using a one-way ANOVA, followed by Tukey’s multiple comparison test. Different superscript letters (a–d) indicate statistically significant differences among groups (*p* < 0.05). Abbreviations: AMPK, AMP-activated protein kinase; p-, phosphorylated; t-, total; PPARα, peroxisome proliferator-activated receptor alpha; SREBP1c, sterol regulatory element-binding protein 1c; ACC, acetyl-CoA carboxylase; CPT1, carnitine palmitoyltransferase 1; FAS, fatty acid synthase; IRS1, insulin receptor substrate 1; Akt, protein kinase B; GLUT2, glucose transporter 2; NRF2, nuclear factor erythroid 2-related factor 2; HO-1, heme oxygenase-1; NQO1, NAD(P)H quinone oxidoreductase 1; GCLC, glutamate–cysteine ligase catalytic subunit; GAPDH, glyceraldehyde-3-phosphate dehydrogenase.

A Western blot analysis showed that F1 treatment markedly increased AMPK phosphorylation (p-AMPK/t-AMPK) in a dose-dependent manner compared with the CON group. Consistent with AMPK activation, the expression of PPARα and CPT1, which are involved in fatty acid oxidation, was significantly upregulated, whereas lipogenic markers such as SREBP1c and FAS were markedly downregulated. In addition, the phosphorylation of ACC (p-ACC/t-ACC) was increased, indicating the inhibition of ACC activity and the suppression of de novo lipogenesis. A quantitative densitometric analysis confirmed these trends, suggesting that F1 suppresses lipogenesis and promotes fatty acid oxidation through AMPK-mediated metabolic regulation (Figure 4A,D).

#### 3.4.2. F1 Improves Insulin Signaling

The phosphorylation of IRS1 (p-IRS1/t-IRS1) and Akt (p-Akt/t-Akt) was slightly increased by F1 treatment, accompanied by a modest upregulation of GLUT2 protein expression compared to CON. Although these changes did not reach statistical significance at all concentrations, the observed trend indicates that F1 may facilitate glucose uptake by enhancing insulin signaling (Figure 4B,E).

#### 3.4.3. F1 Promotes NRF2-Mediated Antioxidant Responses

F1 treatment significantly increased the nuclear accumulation of NRF2 in a concentration-dependent manner, with a concomitant decrease in cytosolic NRF2 levels. In parallel, the protein expression of NRF2 downstream antioxidant enzymes, HO-1 and NQO1, was upregulated in the F1-treated groups compared to the CON group. These findings indicate that F1 activates the NRF2 pathway, thereby enhancing antioxidant defense mechanisms (Figure 4C,E).

#### 3.4.4. F1 Regulates the Expression of Genes Involved in Lipid Metabolism and Antioxidant Defense

A qPCR analysis revealed that F1 significantly reduced mRNA levels of SREBP1c, FAS, and ACC while increasing PPARα expression compared to CON. Furthermore, F1 markedly elevated HO-1, NRF2, and GCLC mRNA expression in a dose-dependent manner. GLUT2 mRNA levels were also increased, particularly by the highest F1 concentration (Figure 4F). These transcriptional changes are consistent with the observed protein expression patterns, supporting the role of F1 in concurrently regulating lipid metabolism and antioxidant defense.

## 4. Discussion

The chronic consumption of HFDs is closely associated with obesity, insulin resistance, and liver dysfunction, conditions collectively driving the global rise of MAFLD [2,36,37]. A prolonged nutritional imbalance further promotes oxidative stress, hepatic lipid accumulation, and systemic inflammation, reflecting the pathophysiological continuum observed in clinical MAFLD. Experimental models ranging from HFD to HFHC regimens are therefore widely applied to evaluate candidate interventions for metabolic liver disease [24,37]. These models reproduce critical clinical features of human MAFLD, including hepatic steatosis, dyslipidemia, and insulin resistance, and thus provide an essential translational platform for testing therapeutic agents [38,39]. In the present study, the HFHC model was employed to recapitulate the multifactorial pathogenesis of MAFLD. Within this model, an excessive lipid intake induced hepatic steatosis, while additional carbohydrate supplementation enhanced de novo lipogenesis, increased oxidative burden, and disrupted insulin signaling, ultimately accelerating disease progression [1,24]. These metabolic alterations mimic the complex interplay of dietary and hormonal factors contributing to human MAFLD, particularly in individuals with obesity or type 2 diabetes.

Against this pathological background, particular emphasis was placed on evaluating the hepatoprotective and metabolic regulatory effects of SGB121 [9], a ginsenoside F1-enriched extract with the rare minor ginsenoside F1. The administration of SGB121 significantly ameliorated HFHC diet-induced alterations in body composition and metabolic parameters. It suppressed excessive body weight gain, reduced fat accumulation, and preserved lean mass, as confirmed by DEXA and relative tissue weight analyses. These improvements parallel clinically relevant outcomes such as enhanced insulin sensitivity and a reduced hepatic lipid burden. Similarly to the findings of Hou et al., who demonstrated that an F1-enriched ginsenoside mixture attenuated HFHC diet-induced tissue injury by inhibiting autophagy-mediated senescence, inflammation, and apoptosis, SGB121 exerted multi-organ protective effects. Furthermore, previous research reported that SGB121 ameliorated scopolamine-induced neuronal and cognitive dysfunction by restoring mitochondrial activity and cellular signaling [8]. Taken together, these findings suggest that SGB121, through its pleiotropic metabolic and antioxidant actions, may represent a safe and multifunctional nutraceutical candidate for mitigating diet-induced metabolic dysfunction. From a clinical perspective, such a compound could complement existing therapeutic strategies targeting insulin resistance, oxidative stress, and hepatic lipid accumulation, which remain central challenges in MAFLD management.

The HFHC diet also induced hepatotoxicity, as evidenced by elevated serum ALT and AST activities, and the extensive accumulation of large lipid droplets in histological analyses [40]. In this study, the absolute liver weight significantly increased in the HFHC-fed mice; however, because body weight increased to a much greater extent, the liver-to-body weight ratio (relative liver weight) appeared to be reduced. This pattern is consistent with that in previous reports. Nishikawa et al. [41] demonstrated that, in C57BL/6J mice fed a high-fat diet, “the relative liver weight decreased because the liver weight was unchanged while body weight markedly increased.” Similarly, Sugimoto et al. [42] reported a significant reduction in the relative liver weight of high-fat diet-fed mice compared with that of controls. Some studies have also noted that prolonged lipid overload and metabolic stress can induce hepatocellular loss or tissue atrophy, which further contributes to reductions in the relative liver weight [43]. Therefore, the seemingly paradoxical decrease in the relative liver mass observed in our study is likely attributable not to the absence of steatosis but rather to a combination of accelerated body weight gain (denominator effect) and hepatocellular atrophy induced by chronic lipotoxic and oxidative stress. These findings underscore the importance of interpreting the relative liver weight in conjunction with both the absolute liver weight and overall body weight, particularly in long-term or severe HFHC feeding models.

Hepatic and circulating TG and TC levels were significantly elevated, confirming systemic dyslipidemia. These alterations align with the canonical mechanisms of MAFLD progression, which involve excessive triglyceride synthesis, the impaired secretion of very-low-density lipoproteins (VLDLs), and diminished β-oxidation [24,44,45]. Furthermore, the excessive lipid burden impaired mitochondrial function and increased ROS generation, leading to elevated MDA and depleted GSH levels. This imbalance between pro-oxidant and antioxidant systems is a well-recognized trigger of hepatocellular injury, inflammation, and apoptosis, facilitating the transition from simple steatosis to MASH [45,46].

The present study provides comprehensive mechanistic and translational evidence supporting the therapeutic potential of SGB121 for MAFLD. In vivo, SGB121 administration ameliorated HFHC diet-induced hepatic steatosis, restored relative liver weight, suppressed large lipid droplet formation, and improved systemic metabolic homeostasis. These hepatoprotective actions were accompanied by reductions in oxidative stress markers such as MDA and the restoration of the antioxidant GSH pool, reflecting the recovery of hepatic redox balance [47]. Consistent with prior findings on *Panax notoginseng* saponins and other minor ginsenosides such as F2 [24,48], SGB121 demonstrated multifaceted regulation of lipid metabolism, oxidative stress, and inflammatory responses, thereby halting MAFLD progression through the dual modulation of lipid deposition and antioxidant defense mechanisms. At the cellular level, FFA-treated HepG2 cells reproduced the key molecular signatures of MAFLD, including lipid droplet accumulation, impaired AMPK activation, diminished PPARα-mediated β-oxidation, and SREBP1-driven lipogenesis [46,49,50].

Within this context, SGB121 and its major constituent ginsenoside F1 demonstrated a coordinated regulation of AMPK-dependent pathways. F1 restored AMPK phosphorylation, enhanced PPARα and CPT1 expression, and concurrently suppressed SREBP1, ACC, and FAS, thereby promoting fatty acid oxidation while inhibiting de novo lipogenesis [36,51,52,53,54]. Moreover, F1 reactivated insulin signaling by restoring IRS–Akt–GLUT2 expression, reinforcing glucose utilization and energy homeostasis. The simultaneous attenuation of oxidative stress and restoration of lipid glucose metabolic signaling illustrates that SGB121 targets multiple pathogenic nodes of MAFLD rather than a single pathway.

These in vitro findings mechanistically complement the in vivo outcomes, revealing a consistent AMPK-centered regulatory network that bridges hepatic cellular protection to systemic metabolic recovery. Notably, SGB121 reduced fasting insulin and HOMA-IR values and modestly improved fasting glucose levels, effects that closely parallel those of metformin, a clinically established insulin sensitizer that acts through AMPK activation and lipogenesis suppression [28,29,30]. This convergence underscores the translational relevance of SGB121, suggesting that ginseng-derived multi-component formulations could emulate or augment metformin-like benefits while potentially offering improved tolerability and safety for the long-term management of MAFLD and associated metabolic disorders.

The complex phytochemical matrix of SGB121 appears to contribute to its broad efficacy through additive or synergistic interactions among ginsenosides such as F1, Rg1, Rb1, and compound K [31,32,33,36,37]. Although purified F1 exhibited potent antioxidant and insulin-sensitizing effects, its concentration within SGB121 was below the pharmacologically effective range, suggesting that the extract’s therapeutic benefits arise from multi-constituent synergy rather than a single compound. Such interactions within the phytochemical matrix may enhance the bioavailability, stability, and cellular uptake of ginsenosides, thereby amplifying their therapeutic potency in both experimental and physiological settings.

These results demonstrate that SGB121 exerts integrated protective effects across molecular, cellular, and systemic levels by normalizing AMPK-dependent lipid redox signaling, restoring insulin sensitivity, and reinforcing antioxidant defense. These mechanisms are directly relevant to the clinical pathology of MAFLD, where dysregulated lipid metabolism, oxidative imbalance, and insulin resistance form an interdependent triad driving disease progression. From a clinical perspective, SGB121 represents a promising nutraceutical candidate that could complement existing pharmacotherapies or serve as a preventive intervention for individuals at risk of hepatic disorders associated with metabolic syndrome. Moreover, the mechanisms identified in this study—the activation of AMPK and PPARα, the suppression of SREBP1-mediated lipogenesis, and the restoration of insulin signaling—closely parallel the pharmacological targets of existing insulin sensitizers and lipid-lowering agents such as metformin and pioglitazone. This suggests that SGB121 could serve as a clinically relevant complementary approach, offering metabolic benefits through multi-pathway modulation with potentially fewer adverse effects. Given its natural origin, safety profile, and oral bioavailability, SGB121 may be applicable as a long-term nutraceutical for the prevention or adjunct management of MAFLD in individuals with metabolic syndrome or prediabetic conditions.

While SGB121 demonstrated promising multi-level efficacy, several limitations should be acknowledged. This study employed a single animal model (HFHC diet-induced MAFLD in C57BL/6J mice) with one dosing regimen, and mechanistic analyses were based primarily on surrogate biomarkers. Future investigations encompassing different metabolic phenotypes, sex-based variations, gut–liver axis interactions, and both pharmacokinetic and safety evaluations will be essential to establish the clinical applicability of SGB121 as a safe and multifunctional agent linking dietary prevention with pharmacological therapy for MAFLD.

## 5. Conclusions

The present study provides comprehensive evidence that SGB121, a ginsenoside F1-enriched extract, exerts broad hepatoprotective and metabolic benefits against HFHC diet-induced MAFLD. In vivo, SGB121 effectively attenuated body weight gain and fat accumulation, improved hepatic function, normalized systemic and hepatic lipid metabolism, and reinforced antioxidant defense by reducing MDA and restoring GSH levels. These effects were accompanied by significant improvements in insulin resistance and glucose homeostasis, comparable to those achieved with metformin. At the cellular level, mechanistic studies revealed that SGB121 and its abundant component F1 mitigate FFA-induced steatosis and oxidative stress by activating AMPK PPARα CPT-mediated β-oxidation, suppressing SREBP1-ACC–FAS-driven lipogenesis, and restoring IRS Akt GLUT2 signaling, thereby improving insulin sensitivity. While the multi-component nature of SGB121 precludes attributing its efficacy solely to F1, the mechanistic evaluation of F1 underscores its pivotal role in regulating lipid metabolism, oxidative stress, and insulin signaling. Collectively, these findings demonstrate that SGB121 exerts multifunctional protective effects through both systemic and cellular pathways, highlighting its potential as a promising therapeutic candidate for the prevention and management of MAFLD and related metabolic disorders.

## Figures and Tables

**Table 1:** Effects of SGB121 on hepatic and serum lipid profiles and liver function markers in high-fat, high-carbohydrate (HFHC) diet-induced MAFLD mice. Hepatic triglyceride (TG), serum TG, hepatic total cholesterol (TC), serum TC, alanine aminotransferase (ALT), and aspartate aminotransferase (AST) levels were measured after 12 weeks of treatment with SGB121 (50, 100, or 200 mg/kg) or metformin (MET, 150 mg/kg). NOR, normal diet group; CON, control group; MET, metformin-treated group.

	NOR	CON	MET	SGB121 (mg/kg)		
				50	100	200
**Hepatic TG (mg/g liver)**	6.7 ± 0.6 ^a^	62.8 ± 7.6 ^b^	36.6 ± 4.9 ^c^	48.4 ± 3.8 ^d^	49.8 ± 5.3 ^d^	27.9 ± 4.9 ^c^
**Serum TG** **(mg/dL)**	46.4 ± 9.4 ^a^	101.5 ± 2.9 ^b^	51.8 ± 7.3 ^c^	85.4 ± 10.7 ^d^	76.8 ± 14.0 ^cd^	76.9 ± 7.4 ^cd^
**Hepatic TC** **(mg/g liver)**	3.0 ± 0.2 ^a^	8.17 ± 1.07 ^b^	5.15 ± 0.5 ^c^	7.07 ± 0.80 ^b^	6.22 ± 0.64 ^b^	3.74 ± 0.30 ^c^
**Serum TC** **(mg/dL)**	152.8 ± 5.7 ^a^	242.3 ± 13.5 ^b^	215.9 ± 6.8 ^c^	227.4 ± 9.4 ^b^	238.5 ± 13.3 ^bc^	203.9 ± 11.3 ^c^
**ALT** **(unit/mL)**	32.3 ± 4.5 ^a^	61.0 ± 7.0 ^b^	41.2 ± 5.0 ^c^	56.2 ± 6.5 ^b^	52.4 ± 5.1 ^bc^	53.8 ± 5.3 ^b^
**AST** **(unit/mL)**	61.6 ± 6.7 ^a^	91.3 ± 8.0 ^b^	67.2 ± 3.5 ^a^	113.4 ± 4.5 ^d^	100.0 ± 19.0 ^b^	71.1 ± 9.5 ^a^

Data are expressed as the mean ± SE (*n* = 8). Statistical significance was analyzed using a one-way ANOVA, followed by Tukey’s multiple comparison test. Different superscript letters indicate statistically significant differences among groups (*p* < 0.05).

## Data Availability

The datasets used and/or analyzed during the current study available from the corresponding.

## Supplementary Materials

The following supporting information can be downloaded at: https://www.mdpi.com/article/10.3390/nu17233693/s1, Table S1. Summary of the preparation process and HPLC analytical conditions used for the standardization of SGB121, a ginsenoside F1–enriched extract produced via microbial biotransformation using β-glucosidase–expressing Corynebacterium glutamicum (BD001); Table S2. Comparative composition and nutritional characteristics of two commonly used rodent diets; Figure S1. HPLC analysis of the transformation of ginsenosides using immobilized C3a-MT619; Figure S2. Representative HPLC chromatograms showing the ginsenoside F1 peak isolated from the standardized extract (SGB121).

## Abbreviations

The following abbreviations are used in this manuscript:

MAFLD	metabolic dysfunction-associated fatty liver disease
NAFLD	non-alcoholic fatty liver disease
NASH	non-alcoholic steatohepatitis
PPT	protopanaxatriol
HFD	high-fat diet
HFHC	high-fat, high-carbohydrate
BSA	bovine serum albumin
DMSO	dimethyl sulfoxide
MTT	3-(4,5-dimethylthiazol-2-yl)-2,5-diphenyltetrazolium bromide
HRP	horseradish peroxidase
DEXA	dual-energy X-ray absorptiometry
MDA	malondialdehyde
TBA	thiobarbituric acid
GSH	glutathione
TG	triglyceride
TC	total cholesterol
AST	aspartate amino transferase
ALT	alanine aminotransferase
HOMA-IR	homeostasis model assessment of insulin resistance
DCFH-DA	2′,7′-dichlorodihydrofluorescein diacetate
