# Supplementary material for: Protective Role of Ginsenoside F1-Enriched Extract (SGB121) in Metabolic Dysfunction-Associated Fatty Liver Disease (MAFLD)
Source: Nutrients. 2025 Nov 25;17(23):3693. doi: 10.3390/nu17233693 (PMC12693820; doi:10.3390/nu17233693)
Supplement: Supplementary file 1 [file nutrients-17-03693-s001.zip › nutrients-3937973-supplementary.pdf]

Table S1. Summary of the preparation process and HPLC analytical conditions used for the standardization of SGB121, a ginsenoside F1–enriched extract.

Category	Description
**Sample Name**	SGB121
**Manufacturer**	ISBC, Daejeon, Korea
**Standardization**	Ginsenoside F1
**Analytical Equipment**	Agilent 1260 series HPLC system with UV detector (Agilent Technologies, Palo Alto, CA, USA)
**Analytical Column**	YMC-Triart C18 (4.6 × 250 mm, 5 μm; YMC Co., Kyoto, Japan)
**Mobile Phase**	Solvent A: Water; Solvent B: Acetonitrile
**Flow Rate**	1.6 mL/min
**Injection Volume**	30 μL
**Detection Wavelength**	203 nm
**Gradient Elution Program**	0–10 min: 20% B; 10–40 min: 32% B; 40–48 min: 42% B; 48–60 min: 45% B; 60–83 min: 75% B; 85–95 min: 100% B; 95–95.01 min: 20% B; 95.01–100 min: 20% B

Previously published dataset [1,2] was re-analyzed under the current experimental framework.”

Supplementary Table S2. Comparative composition and nutritional characteristics of two commonly used rodent diets. The Research Diets D12091402 formulation is a purified high-fat (60 kcal% fat) diet typically used to induce obesity and metabolic syndrome in rodents, whereas the Teklad Global 18% Protein Rodent Diet (2918) is a standard grain-based maintenance diet used as a normal-control feed. Marked differences exist in energy density, macronutrient distribution, and fat/carbohydrate sources, which should be considered when designing comparative in vivo studies.

Category	Research Diets D12091402 (60 kcal% Fat, DIO Purified Diet)	Teklad Global 18% Protein Rodent Diet (2918, Inotiv)
**Diet Type**	Purified diet formulated for diet-induced obesity (DIO) models	Natural ingredient diet for maintenance and growth
**Energy Density**	5.21 kcal/g	3.1 kcal/g
**Energy Distribution (kcal%)**	Protein 20 % / Fat 60 % / Carbohydrate 20 %	Protein 24 % / Fat 18 % / Carbohydrate 58 %
**Major Protein Source**	Casein (Lactic, 30 Mesh) + L-Cystine	Soybean meal, wheat middlings, ground corn (mixed plant proteins)
**Major Carbohydrate Source**	Fructose (100 g), Lodex 10 (93.8 g), Sucrose (4 g)	Wheat middlings and ground corn (starch-based polysaccharides)
**Fat Source**	Lard (245 g) + Soybean oil (25 g)	Vegetable oils (mainly soybean and corn lipids; ether extract 6.2 %)
**Fiber Source**	Solka Floc (FCC200, 50 g cellulose)	Mixed natural fibers (cellulose and grain fiber)
**Vitamin Mixture**	V10001C (Vitamin Mix) + Choline Bitartrate (2 g)	Vitamins A (15 IU/g), D_3 (1.5 IU/g), E (11 IU/g), K, B-complex, folic acid
**Mineral Mixture**	S10026B (Mineral Mix, 50 g)	Ca 1.0 %, P 0.7 %, K 0.8 %, Na 0.3 %, Fe 200 mg/kg (grain-derived minerals)
**Representative Amino Acids**	Derived from casein (supplemented with L-Cystine)	Aspartic 3.4 %, Glutamic 6.1 %, Leucine 1.8 %, Lysine 1.0 %
**Representative Fatty Acids**	Mainly from lard (saturated and monounsaturated fats)	C16:0 (0.7 %), C18:0 (0.2 %), C18:1 (1.2 %), C18:2 (1.3 %) → Total PUFA 3.4 %
**Physical Form**	Purified powder/pellet (dry, non-irradiated)	Irradiated pellet (stable for ≤ 9 months under proper storage)
**Primary Purpose**	Induction of obesity, hepatic steatosis, and metabolic dysfunction	Standard maintenance, growth, and reproductive studies
**Notable Features**	High fat content with fructose carbohydrates → effective for metabolic stress induction	Alfalfa-free composition → low background autofluorescence for imaging studies

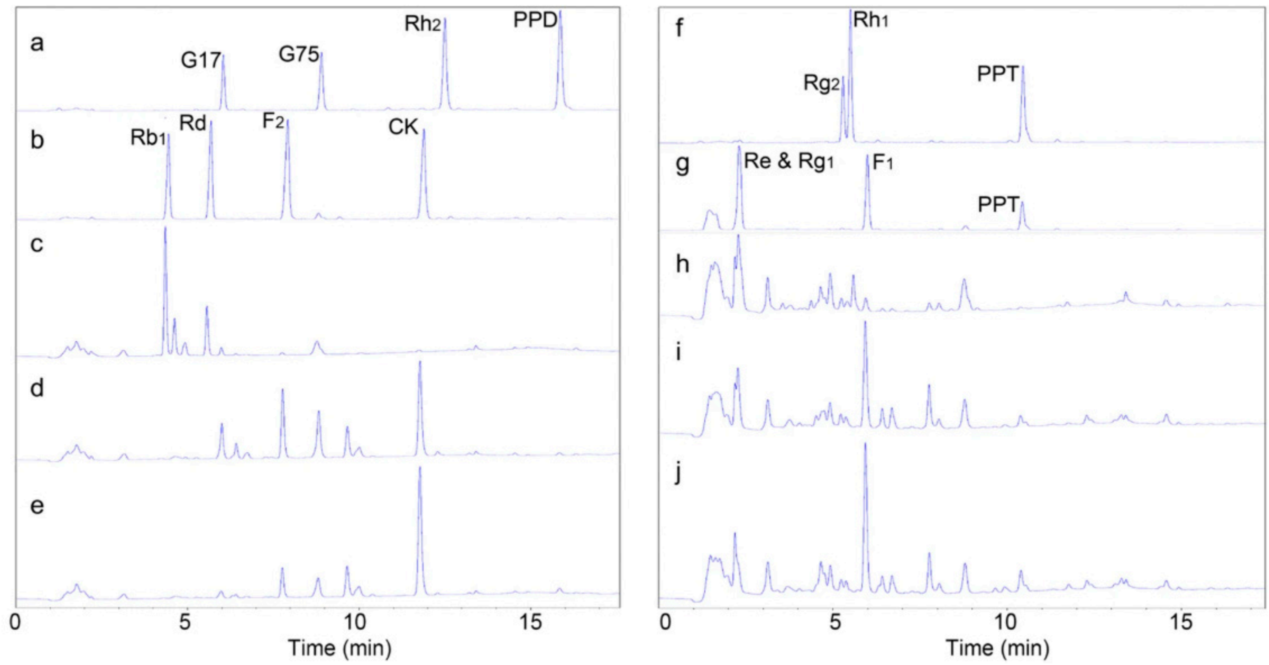

Supplementary Figure S1. HPLC analysis of the transformation of ginsenosides using immobilized C3a-MT619. **a** Ginsenoside standards (G17, G75, Rh_2, PPD). **b** Ginsenoside standards (Rb_1, Rd, F_2, CK). **c** PPDGM. **d** CK produced after 6 h of reaction of immobilized C3a-MT619 with PPDGM. **e** CK produced after 24 h of reaction. **f** Ginsenoside standards (Rg_2, Rh_1, PPT). **g** Ginsenoside standards (Re, Rg_1, F_1, PPT). **h** PPTGM. **i** F_1 produced after 6 h of reaction of immobilized C3a-MT619 with PPTGM. **j** F_1 produced after 24 h.

This chromatogram was redrawn from previously published data [2] to illustrate the specific elution profile of F1 under standardized quantification conditions.

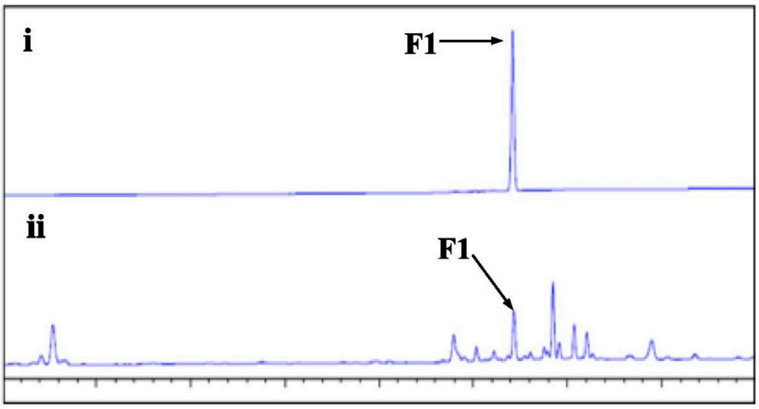

Supplementary Figure S2. Representative HPLC chromatograms showing the ginsenoside F1 peak isolated from the standardized extract (SGB121). (i) Purified ginsenoside F1 standard analyzed under identical chromatographic conditions. (ii) SGB121 extract containing ginsenoside F1 (0.5% w/w) analyzed by HPLC using an Agilent 1260 system equipped with a YMC-Triart C18 column (4.6 × 250 mm, 5 μm) and UV detection at 203 nm. The F1 peak was detected at the same retention time as the purified standard, confirming the successful enrichment of ginsenoside F1 in SGB121.

This chromatogram was redrawn from previously published data [1] to illustrate the specific elution profile of F1 under standardized quantification conditions.

Reference

1. Bastola, T.; Pariyar, R.; Jeon, B.-M.; Baek, J.-I.; Chang, B.Y.; Kim, S.-C.; Kim, S.Y.; Seo, J. Protective effects of SGB121, ginsenoside F1-enriched ginseng extract, on scopolamine-induced cytotoxicity and memory impairments. *Journal of Functional Foods* **2020**, *74*, 104165, doi:https://doi.org/10.1016/j.jff.2020.104165.
2. Cui, C.-h.; Jeon, B.-M.; Fu, Y.; Im, W.-T.; Kim, S.-C. High-density immobilization of a ginsenoside-transforming β-glucosidase for enhanced food-grade production of minor ginsenosides. *Applied Microbiology and Biotechnology* **2019**, *103*, 7003-7015, doi:10.1007/s00253-019-09951-4.
